# Atomic Layer Deposited Hf_0.5_Zr_0.5_O_2_-based Flexible Memristor with Short/Long-Term Synaptic Plasticity

**DOI:** 10.1186/s11671-019-2933-y

**Published:** 2019-03-15

**Authors:** Tian-Yu Wang, Jia-Lin Meng, Zhen-Yu He, Lin Chen, Hao Zhu, Qing-Qing Sun, Shi-Jin Ding, David Wei Zhang

**Affiliations:** 1 State Key Laboratory of ASIC and System, School of Microelectronics, Fudan University, Shanghai, 200433 China; 2 Frontier Science Center for Synthetic Biology and Key Laboratory of Systems Bioengineering (MOE), and School of Chemical Engineering and Technology, Tianjin University, Tianjin, 300072 China

**Keywords:** Atomic layer deposition, Low-temperature process, Flexible electronics, Synaptic plasticity

## Abstract

Artificial synapses are the fundamental of building a neuron network for neuromorphic computing to overcome the bottleneck of the von Neumann system. Based on a low-temperature atomic layer deposition process, a flexible electrical synapse was proposed and showed bipolar resistive switching characteristics. With the formation and rupture of ions conductive filaments path, the conductance was modulated gradually. Under a series of pre-synaptic spikes, the device successfully emulated remarkable short-term plasticity, long-term plasticity, and forgetting behaviors. Therefore, memory and learning ability were integrated to the single flexible memristor, which are promising for the next-generation of artificial neuromorphic computing systems.

## Background

The classical von Neumann computing scheme is suffering a bottleneck of information transfer between the processing center and storage units [1]. Through emulating biological brains, neuromorphic computing has become an attractive candidate with the ability of learning and memory in one single system [2, 3]. Electronic synapses, with the ability of mimicking bio-synaptic behavior, are the foundation of neuromorphic systems. Recently, bio-synaptic behaviors have been emulated by various memristors, including two-terminal devices and novel three-terminal synaptic transistors based on ionic defects [4, 5]. With history-dependent conductance, memristors were reported to simulate the long-term depression (LTD) or potentiation (LTP), pair-pulse fluctuation (PPF), paired-pulse depression (PPD), and spike-timing-dependent plasticity (STDP) [6–8]. Especially, LTP/LTD is vital for face classification, digital recognition, and other artificial intelligence applications based on synaptic weight modification [9–11]. Originating from immediate post-synaptic current response, STP is widely used for information filtering and instantaneous signal transmission [12].

A variety of material systems were studied for artificial synapses with bio-synaptic plasticity, including HfO_2_, ZnO, WO_x_, TaO_x_, InGaZnO, organic polymers, and 2D transition-metal dichalcogenides (TMDCs) [13–19]. Among them, Hf_0.5_Zr_0.5_O_2_ (HZO) is one of the novel high-k materials and compatible with the process of complementary metal oxide semiconductor (CMOS) [20]. Although HZO-based artifical synapstic devices have been reported, the high-temperature preparation process is hard to aviod [21–23].

On the other hand, flexible artificial synaptic devices were widely studied to satisfy the rising need for wearable artificial intelligence applications [24, 25]. However, the high-temperature preparation process is an impediment to the application of a flexible substrate. Although a transfer process was proposed to solve the problem, the high failure rate and wrinkle defects caused by transfer hinder the large-scale use of this method [26, 27]. It is worth noting that low-temperature processing has no damage to flexible substrates, which is an effective way of developing large-scale wearable synaptic arrays.

In this work, a low-temperature ALD technique for HZO-based memristor (PET/ITO/HZO/Ag) was developed. Gradual conductance switching process was demonstrated in this memristor. Based on gradual resistance switching characteristics, typical synaptic plasticity was emulated, including LTP/LTD, STP, PPF, and forgetting curves. With the function of biological synapses, the flexible HZO-based memristor is attractive for future applications in a neuromorphic computing system.

## Methods

**Fig. 1:**
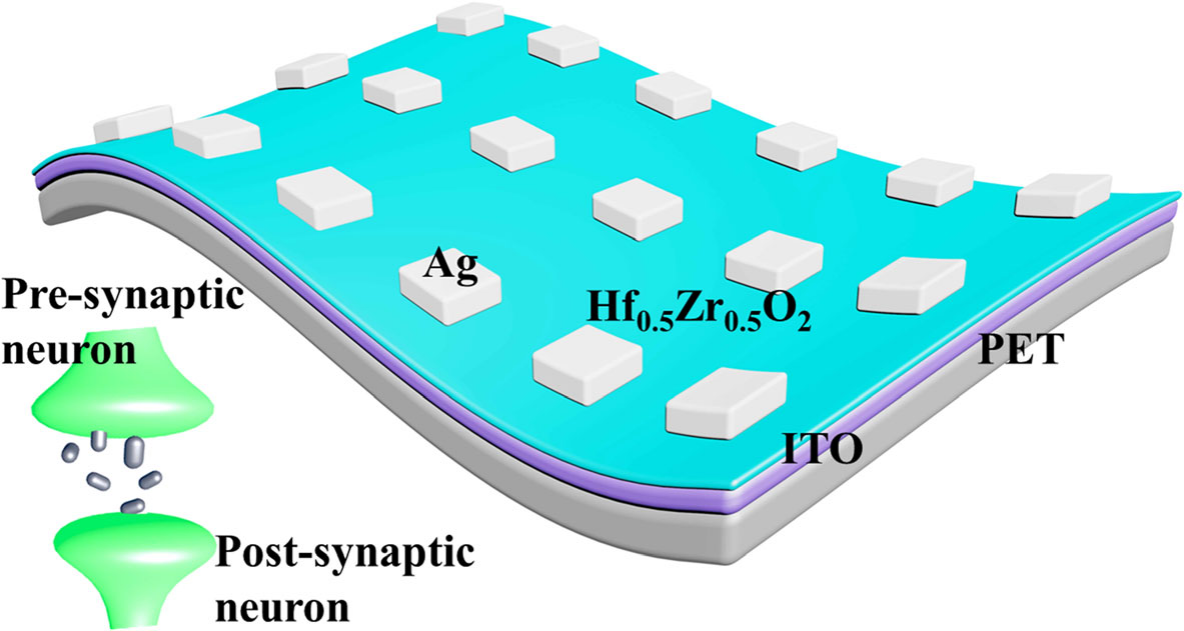
Schematic illustration of biological synapse between neurons and artificial electrical synapses. A bio-synapse was composed of pre-synaptic neuron, synaptic cleft, and post-synaptic neuron. The HZO-based flexible electrical synapse was fabricated with the structure of ITO/HZO/Ag on the plastic substrate at low temperature

The flexible synaptic device was prepared on ITO-coated polyethylene terephthalate (PET) substrate, which was cleaned in acetone, isopropanol, and deionized water and dried by N_2_ flow. A 10-nm-thick HZO film was deposited on PET/ITO substrate by ALD with the carrier gas of N_2_. The precursors were tetrakis (ethylmethylamino) hafnium (TEMAH), tetrakis (ethylmethylamino) zirconium (TEMAZ), and H_2_O, and the growth temperature of the ALD chamber was maintained at 130 °C. Then, a 50-nm Ag top electrode (TE) layer with an area of 100 × 100 μm^2^ was deposited by physical vapor deposition (PVD) followed by photolithography and lift-off process. The structure of PET/ITO/ HZO/Ag was shown in Fig. 1. The top electrode of Ag and bottom electrode of ITO are corresponding to pre- and post-synaptic neuron in biological synapse.

The electrical characteristics were performed using a semiconductor parameter analyzer (Agilent B1500A) in the atmospheric environment at room temperature. The bottom electrode was grounded while the programming bias was applied to the top electrode.

## Results and Discussion

**Fig. 2:**
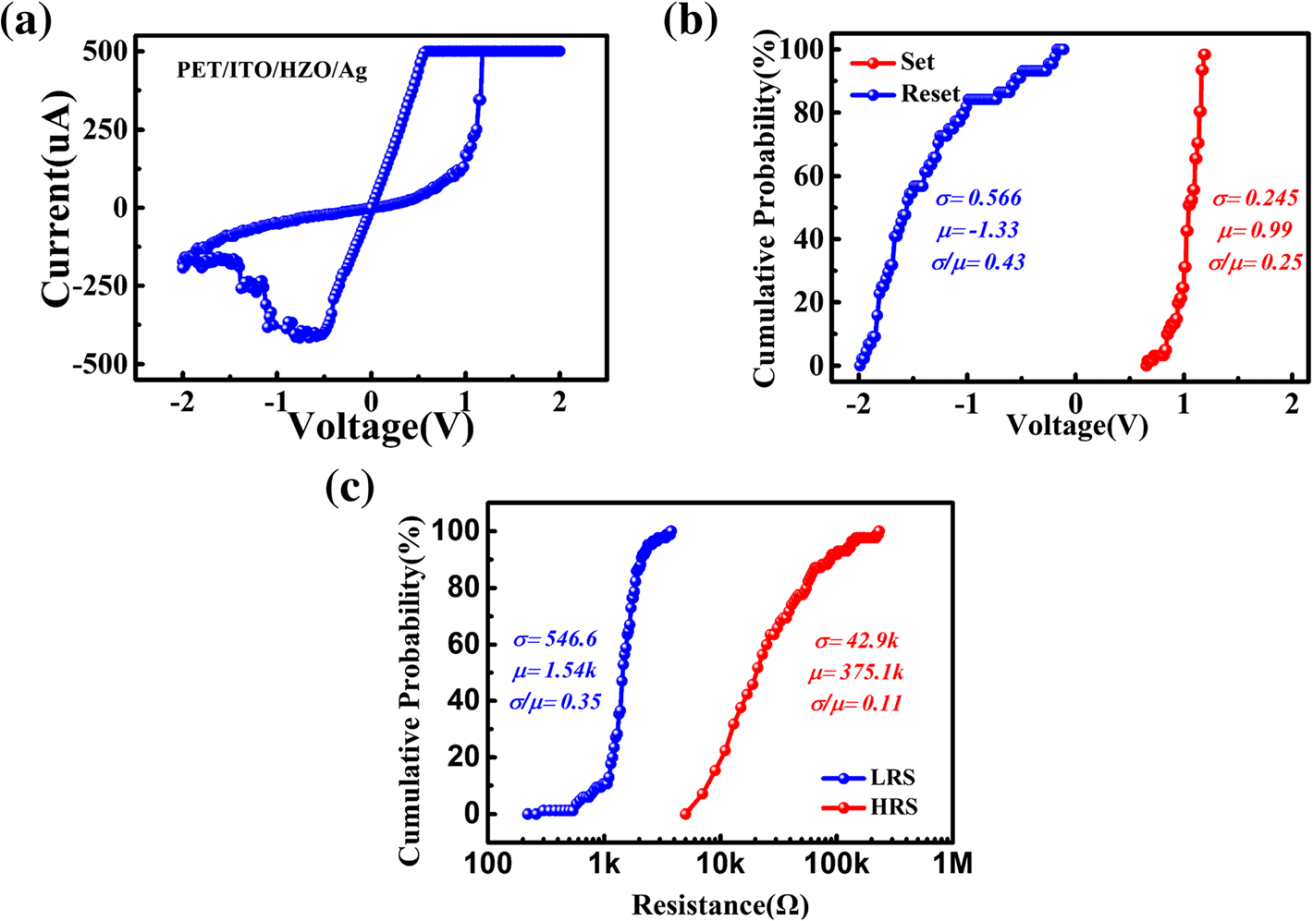
**a** Resistive switching characteristics of HZO-based device measured by DC sweep. **b** Distribution of the set and reset voltages extracted from DC sweep cycles in flexible device. **c** Statistical data of HRS and LRS, where resistance were measured at a read voltage of 0.1 V

Figure 2a shows the typical bipolar resistive switching curve of the memristor with the current compliance of 500 uA. The sweeping voltage was applied in a sequence of 0 → 2 V → 0 V for the set process, and the resistance turned from high-resistance state (HRS) to low-resistance state (LRS). In contrast, a negative voltage was applied from 0 V to − 2 V and returned to 0 V for the reset process. The gradual switching characteristic in positive and negative bias sweeps indicates the potential of HZO-based memristor emulating synaptic behaviors. The cumulative probability of operating voltages in the set and reset process during consecutive sweep cycles are shown in Fig. 2. The means (μ) of the set voltage and reset voltage are 0. 99 V and − 1. 33 V, respectively, which showed the average level of operating voltage. The standard deviation (σ) of the operating voltage (0.245 for set process and 0.566 for reset process) indicated the degree of deviation from the center. The relative fluctuation of data could be described as a coefficient of variance (σ/μ). Superior uniformity was obtained in the set process while the variation of HRS resistance and reset voltage are remarkable, which could attribute to the formation and rupture process of conductive filament (CF) of Ag atoms. During the process of set operation, the size or number of CFs would increase. The current level of device is almost linearly proportional to the increment of CFs. During the reset process, the CFs would break and decrease. While the current level of device is exponentially dependent on the breaking length of CFs [28]. A small change of CFs during the reset process could result to obvious changes of resistance and reset voltage. The ON/OFF ratio of μ in HZO-based device was larger than 300, as shown in Fig. 2c.

**Fig. 3:**
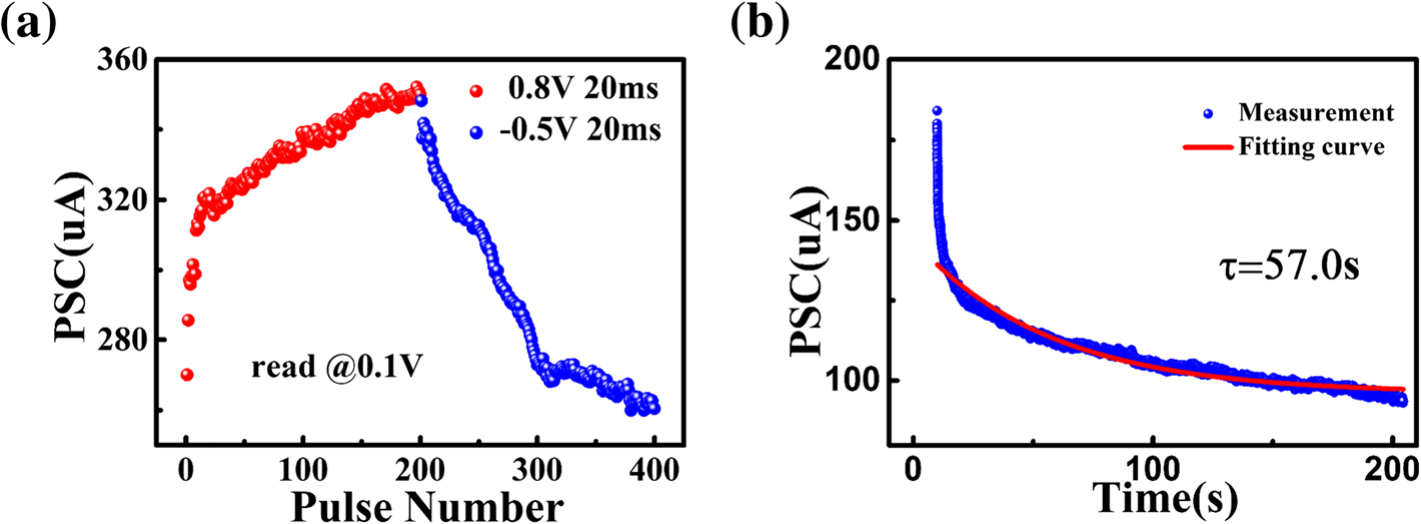
**a** Gradual conductance modulation for LTP and LTD in the artificial flexible synapse, where the post-synaptic current was obtained at a read voltage of 0.1 V. **b** Forgetting behaviors after 100 consecutive programing pulses (1 V, 50 ms) and fitted curves of the electrical synapse

Besides gradually resistance switching behaviors in DC sweep, the device with modulated conductance could be programed by a sequence of consecutive pulses. As shown in Fig. 3a, the conductance could be modulated gradually to emulate LTP and LTD with 400 consecutive programing pulses, indicating the potential of the synaptic device for neuromorphic computing. With 200 consecutive positive pulses (0 .8V, 20 ms) and 200 negative pulses (− 0 .5V, 20 ms), the conductance of synaptic device potentiated and depressed gradually. The conductance state was obtained under a read voltage of 0.1 V after each consecutive pulse. Forgetting is one of the common phenomena in human brains, which could be simulated by the relaxation of post-synaptic current in electrical synapses. After a series of pulses, the post-synaptic current (PSC) decayed and turned to an intermediate state over time, as shown in Fig. 3b. The forgetting curve could be fitted with the Kohlrausch equation that was frequently used in psychology:

1 $$ I(t)={I}_0+A\exp \left(-t/\tau \right) $$

where *I(t)* is the PSC at the time of *t*, *I*_0_ is the stabilized current, A is a prefactor, and *τ* is a relaxation time constant. In the artificial synaptic device, the constant *τ* was 57 s which was used to evaluate the forgetting characteristics.

**Fig. 4:**
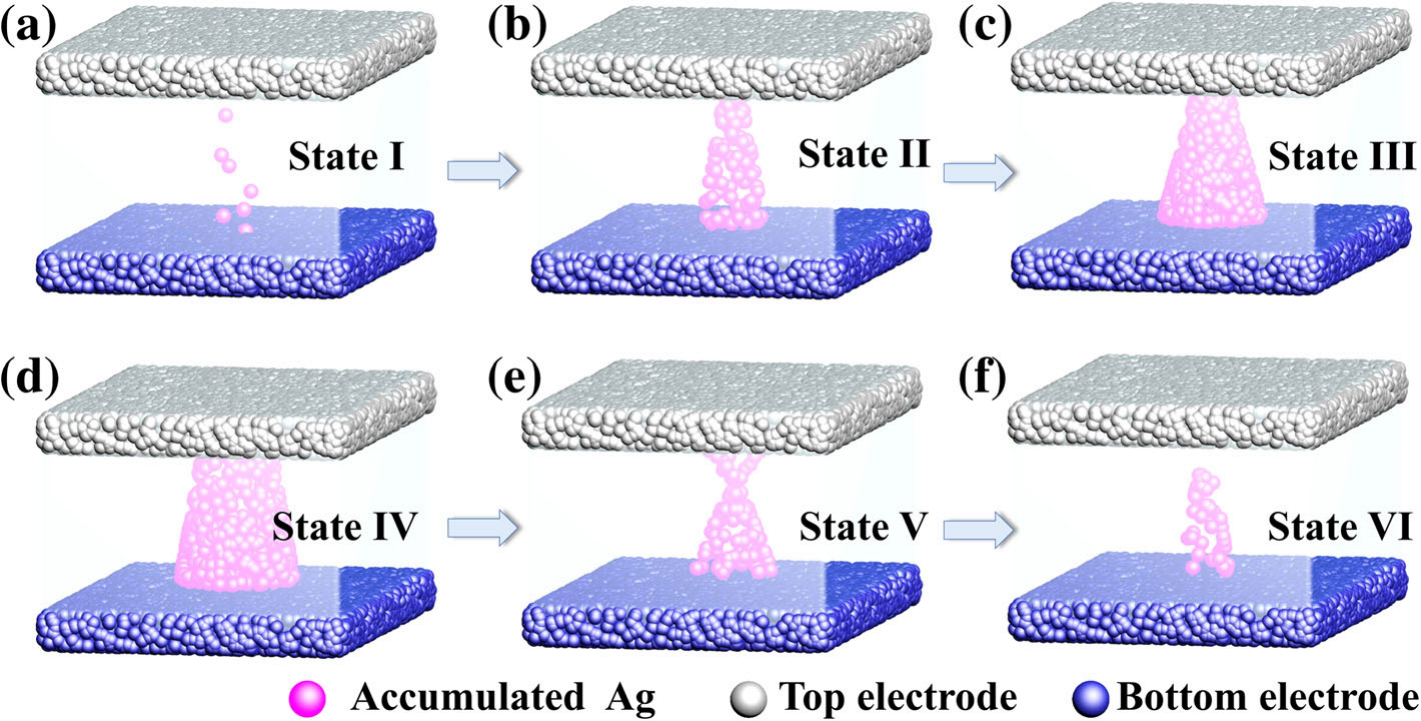
**a**–**c** The schematic diagrams of forming Ag cations conductive path under consecutive positive pulses in LTP. **d**–**f** Rupture of the conductive filament after consecutive negative pulses in LTD

To better understand the work mechanism of the HZO-based synaptic device, the conductive filaments (CF) in different states were shown in Fig. 4. The formation and rupture of the CFs were due to the migration of Ag atoms and mobile Ag^+^. When the positive programing stimulus was applied to the top electrode, atoms of top electrode were oxided to Ag^+^, which were accumulated in the bottom electrode and reduced to Ag atoms. In Fig. 4a–c, the thickness and diameter of CF increased slightly from state I to state III, which induced the conductance increase [29]. In contrast, the bridge of Ag atoms ruptured with a weak effect on the conductance after applying a series of negative spikes in memristor, as shown in Fig. 4d–f. Typical LTP and LTD behaviors in this HZO-based artificial synaptic device were organized from the CF gradual formation and rupture, respectively.

**Fig. 5:**
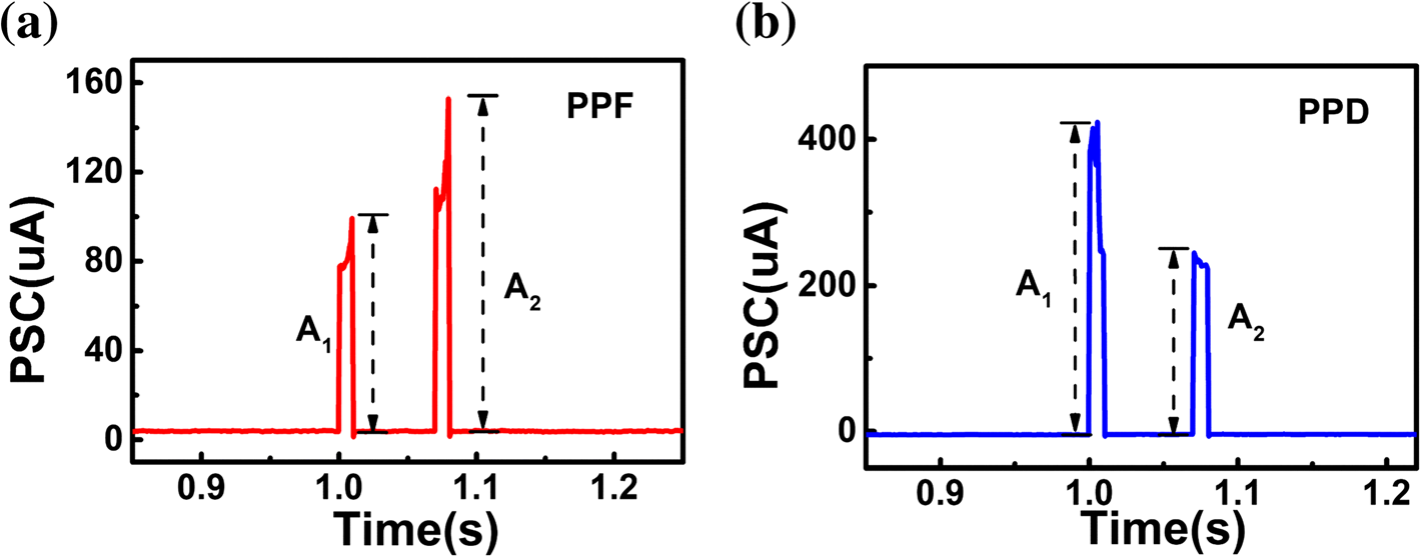
**a** Typical PPF behavior induced by a pair of pre-synaptic spikes (2 V, 10 ms). **b** The PPD phenomenon of the artificial flexible synapse under inhibited spikes (− 1 .5V, 10 ms)

Short-term synaptic plasticity is crucial to both excitatory and inhibitory bio-synapses, which is considered to play important roles in treatment of temporal information [30, 31]. The PPF and PPD behaviors are typical short-term phenomenon organized from two consecutive synaptic spikes with a short interval. Such plasticity was also successfully mimicked in our flexible HZO-based synaptic device. The PPF function was short-term enhancement of synaptic weights trigged by a pair of spikes (2 V, 10 ms) with an interval of 60 ms, as shown in Fig. 5a. In contrast, the response current of second spike is smaller than that of previous spike, which is described as PPD and simulated by two negative pulses (− 1 .5V, 10 ms) with an interval of 60 ms.

**Fig. 6:**
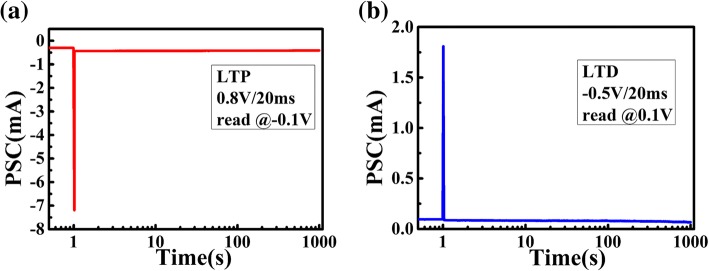
**a** The retention characteristics of electrical synapse under positive programing pulse, indicating the long-term potential behaviors. **b** In LTD process, the post-synaptic current could be inhibited under a single negative pulse (− 0 .5V, 20 ms) and the conductance state could stay stable for over 1000 s

To demonstrate the reliability of long-term plasticity in our synaptic device, retention characteristics were measured for over 1000 s. As shown in Fig. 6, the PSC in excitatory and inhibitory states were read at a bias of 0.1 V after a single pre-synaptic spike. The long-term retention behavior of our HZO-based device shows the potential of storage, and the consecutive modulated conductance paves the way for memory function, which could be integrated into a system.

## Conclusions

In summary, a flexible HZO-based artificial synaptic device was proposed based on low-temperature ALD. Typical bipolar resistive switching characteristics were demonstrated in this flexible memristor. By applying consecutive pulses in the top electrode, long-term plasticity and short-term plasticity were simulated by the electrical synapse, including LTP, LTD, PPF, PPD, and forgetting behaviors. Gradually modulated conductance could be attributed to controllable Ag ions conductive filament path. The flexible electrical synapse becomes one of the promising candidates for hardware implementation of neuromorphic circuits.

## Abbreviations

ALD: Atomic layer deposition
HRS: High-resistance state
LRS: Low-resistance state
LTD: Long-term depression
LTP: Long-term potentiation
STP: Short-term plasticity
